# High-frequency percussive ventilation in pediatric acute respiratory failure in a community-based pediatric intensive care unit

**DOI:** 10.3389/fped.2026.1864661

**Published:** 2026-09-11

**Authors:** Juan Sebastian Proaño, Daniel Jayasuriya, Balagangadhar R. Totapally

**Affiliations:** 1 Department of Pediatrics, Marshall University Joan C Edwards School of Medicine, Huntington, WV, United States; 2 Division of Pediatric Critical Care, Hoops Family Children’s Hospital, Huntington, WV, United States; 3 Division of Internal Medicine, Cabell Huntington Hospital, Huntington, WV, United States; 4 Division of Critical Care Medicine, Nicklaus Children’s Hospital, Miami, FL, United States; 5 Herbert Wertheim College of Medicine, Florida International University, Miami, FL, United States

**Keywords:** community hospital, high frequency percussive ventilation, invasive mechanical ventilation, oxygen saturation index (OSI), PARDS

## Abstract

**Background:**

High-frequency percussive ventilation (HFPV) is an alternative mode of mechanical ventilation that combines time-cycled, pressure-controlled breaths with superimposed high-frequency percussions. Pediatric data suggest improved gas exchange with HFPV. Most reports originate from large academic centers with extracorporeal membrane oxygenation (ECMO) capability. We aimed to evaluate HFPV outcomes in children with acute respiratory failure managed in a community-based pediatric ICU without ECMO capability and to determine whether early changes in oxygen saturation index (OSI) are associated with HFPV failure.

**Methods:**

We conducted a single-center retrospective cohort study of intubated children with acute respiratory failure who received HFPV for ≥12 h between 2018 and 2023 in a 10-bed PICU without ECMO capability. HFPV failure was defined as death or transfer for higher-level cardiopulmonary support. Changes in OSI (delta-OSI) were analyzed using receiver operating characteristics (ROC) curves to assess the association of HFPV failure.

**Results:**

Thirty-one subjects met inclusion criteria; median age was 60 months (interquartile range [IQR] 4–180). Seven subjects (23%) met criteria for HFPV failure, including 5 deaths (16%) and 2 transfers (7%) for higher level of cardiopulmonary support. Oxygenation trajectories differed between groups, with higher OSI at 24 h in the failure group (21.7 [IQR 6.4–22.0] vs. 10.3 [IQR 8.0–15.2]). Discriminative ability of delta-OSI improved over time, with the highest area under the curve observed at 24 h.

**Conclusions:**

In a resource-limited PICU without ECMO capability, lack of improvement in OSI within 12–24 h of HFPV initiation was associated with HFPV failure. These findings suggest that early OSI trajectories may aid clinical decision-making regarding escalation of care, though prospective, multicenter validation is needed.

## Introduction

Acute respiratory failure is the leading cause of pediatric intensive care unit (PICU) admission and the most common form of organ failure among critically ill children, with approximately 15%–17% of PICU patients requiring invasive mechanical ventilation (1). As respiratory support practices evolve, alternative modes of ventilation such as high-frequency percussive ventilation (HFPV) have gained attention. HFPV delivers time-cycled, pressure-controlled breaths superimposed with high-frequency percussions throughout the respiratory cycle, creating a hybrid mode that combines features of conventional and high-frequency ventilation (2, 3). HFPV was initially used in patients with burn-related inhalation injury for secretion clearance (3–5). Its use has expanded into pediatric practice, particularly for conditions such as pediatric acute respiratory distress syndrome (PARDS) and bronchiolitis (6–8).

Despite their growing use, alternative ventilatory modes such as HFPV remain understudied in pediatric populations. Most available pediatric HFPV data are derived from large academic centers, leaving limited evidence to guide its use in smaller PICUs that lack advanced cardiopulmonary support. At our community-based pediatric center (9) Cabell Huntington Hospital in West Virginia, HFPV has been used since the early 1990s as an alternative ventilatory strategy for children with acute respiratory failure. In a resource-limited institution with no extracorporeal membrane oxygenation (ECMO) support, such as ours, it is both crucial and time-sensitive to identify patients at risk of HFPV failure to facilitate early transfer to a center with higher-level cardiopulmonary support. We conducted a retrospective analysis of pediatric patients with acute respiratory failure who received HFPV at our institution. Our primary aim was to determine whether changes in oxygenation after initiation of HFPV predict HFPV success or failure in a pediatric center without ECMO capability. Secondary objectives included describing the clinical characteristics, ventilator settings, and outcomes associated with HFPV use in a resource-limited pediatric intensive care setting.

## Methods

### Study design and setting

This was a single-center, retrospective cohort study conducted in the PICU at Hoops Children's Hospital, a 200-bed facility within Cabell Huntington Hospital in West Virginia. It is a resource-limited PICU serving a predominantly rural catchment area. The hospital serves a catchment area of approximately 1.4 million people across 40 counties in West Virginia, Kentucky, and Ohio, with approximately 20% of the population under the age of 17. The 10-bed PICU admits approximately 500 patients annually, including transfers from outlying institutions, and primarily serves a community with a Child Opportunity Index composite score ranging from very low to moderate. Our institution does not have access to ECMO services.

### Participants

The study included intubated pediatric patients who received HFPV for at least 12 h between 2018 and 2023. This minimum duration was chosen to better capture the effect of HFPV on clinical outcomes. HFPV was delivered using the VDR-4 ventilator (Percussionaire, Sandpoint, Idaho). Although the decision to transition to HFPV, as well as other aspects of ventilator management, sedation, and supportive therapies, was not protocolized and was determined at the discretion of the clinical team, our institutional practice is to consider a transition from conventional ventilation to HFPV in children with respiratory failure refractory to conventional strategies. Typical triggers include persistently elevated peak inspiratory pressures (≥32 cm H_2_O), ongoing hypoxemia (SpO_2_ < 88%), and hypercarbia with resultant respiratory acidosis (pH < 7.20) despite optimization of conventional ventilator settings. HFPV is also favored in the setting of significant air-leak syndrome, and in patients with copious secretions refractory to standard pulmonary toilet. The study was approved by the Institutional Review Board (IRB2105483) with a waiver of informed consent.

### Variables and outcomes

The primary outcome was HFPV success or failure, and the primary prognostic variable was the change in oxygen saturation index (delta-OSI) over time. HFPV success was defined as a successful transition from HFPV to a conventional mechanical ventilation or planned extubation. HFPV failure was defined as death or transfer to another institution for higher-level cardiopulmonary support. Patients transferred to another institution for definitive or subspecialty care after transitioning back to conventional mechanical ventilation were not classified as HFPV failures.

We collected conventional ventilator settings 2 h prior to HFPV initiation and HFPV settings 2 h after HFPV initiation. Physiologic and laboratory variables, including SpO_2_, partial pressure of carbon dioxide (PCO_2_), heart rate, mean arterial pressure, central venous pressure, and base excess or deficit, were recorded 2 h before HFPV initiation and at 2, 4, 12, 24, and 48 h after HFPV initiation. Mean airway pressure and FIO_2_ were recorded within the same 60-min interval as SpO_2_ to allow calculation of oxygenation indices, including OSI and S/F ratio. FIO_2_ was recorded and analyzed as a fraction (range 0.21–1.0). When data were not available within that interval, missing values were imputed using a last-observation-carried-forward approach. Carrying values forward was not appropriate in three circumstances: when no preceding value existed to carry forward, when the ventilator setting variable would not be expected to remain equivalent across the transition from conventional ventilation to HFPV, or when the patient had died before the subsequent time point. In these instances, values were treated as missing rather than imputed, and patients were excluded from analyses at that specific time point. Vasoactive infusion score (VIS) was recorded prior to HFPV initiation and at 24 and 48 h after HFPV initiation. Delta OSI was defined as the change from the pre-HFPV OSI measured 2 h before HFPV initiation to OSI values at the predefined post-HFPV time points (2, 4, 12, 24, and 48 h).

Additional data collected included demographic characteristics, discharge diagnoses, Pediatric Index of Mortality 3 (PIM3) score, indication for mechanical ventilation, PICU and hospital length of stay, duration of conventional mechanical ventilation prior to HFPV, duration of HFPV support, reason for HFPV discontinuation, reason for interfacility transfer, discharge status, mortality, air-leak events, and use of adjunctive therapies, including inhaled nitric oxide, systemic steroids, and neuromuscular blockade.

Oxygen saturation index (OSI) and SpO_2_/FiO_2_ ratio (S/F) were calculated only when SpO_2_ was ≤97% (10). OSI was calculated using the following formula: (FiO_2_ × mPaw × 100)/SpO_2_. The VIS was determined using the following formula: (dopamine dose in mcg/kg/min) + (dobutamine dose in mcg/kg/min) + (100 × epinephrine dose in mcg/kg/min) + (100 × norepinephrine dose in mcg/kg/min) + (10 × milrinone dose in mcg/kg/min) + (10,000 × vasopressin dose in units/kg/min) + (100 × phenylephrine dose in mcg/kg/min) (11). PIM3 scores were calculated using the first recorded values after PICU admission (12). Most blood gas measurements were venous. All data were entered into a REDCap database for analysis.

Descriptive statistics were used to summarize the study population. Categorical variables were compared using chi-square analysis or Fisher's exact test, as appropriate, and are presented as percentages with 95% confidence intervals. Continuous variables were compared using the Mann–Whitney *U* test and are presented as medians with interquartile ranges (IQR). Receiver operating characteristic (ROC) analyses were performed to evaluate the predictive value of OSI change (delta-OSI) for HFPV failure at each time point after HFPV initiation. Area under the curve (AUC) values were calculated to assess discriminative ability. A two-sided *P* value < .05 was considered statistically significant. Statistical analyses were performed using SPSS Statistics version 30.0 (IBM Corporation, Armonk, NY).

## Results

Of the 33 patients who received HFPV during the study period, 2 were excluded because they received HFPV for fewer than 12 h. The first excluded patient was an infant admitted after prolonged cardiac arrest who was placed on HFPV due to persistent mixed respiratory and metabolic acidosis and died 8 h after admission, having received 7 h of HFPV. The second excluded patient was a tracheostomy-dependent teenager with a complex past medical history who was admitted for community-acquired pneumonia and placed on HFPV for only 4 h. Of the 31 patients included in the analysis, 2 were transitioned from high-frequency oscillatory ventilation (HFOV) to HFPV, and 4 had received conventional mechanical ventilation for less than 2 h prior to HFPV initiation. Seven patients (23%) met criteria for HFPV failure: 5 (16%) died, and 2 (7%) were transferred for higher-level cardiopulmonary support.

The demographic details of 31 patients in the analytic cohort are presented in Table 1. Median age was 60 months (IQR 4–180), median weight was 20 kg (IQR 9–54), and 17 patients (55%) were male. Most patients were identified as white (90%). Median PIM3 risk of mortality (ROM) was 1.7 (IQR 0.5–4.4), with no significant difference between the two groups (*P* = 0.98).

**Table 1:** Baseline demographic and clinical characteristics of the study cohort stratified by HFPV outcome.

Variable	All (*n* = 31)	HFPV-success (*n* = 24)	HFPV-failure (*n* = 7)	*P* value
Sex, male	17 (55)	11 (46)	6 (86)	.09
Weight, kg	20 (9–54)	21 (8–62)	14 (11–51)	.90
Age (months)	60 (4–180)	60 (4–192)	48 (1–120)	.41
PIM3	1.7 (0.5–4.4)	2.1 (0.525–4.3)	1.2 (0.5–33)	.98
White	28 (90)	22 (92)	6 (86)	.55
Other race, unspecified	3 (10)	2 (8)	1 (14)	.55
PICU LOS, hours	612 (137–695)	663 (542–711)	222 (96–254)	.004
Hospital LOS, hours	645 (296–786)	694 (542–834)	234 (96–296)	.004
Total time on mechanical ventilation, hours	416 (285–554)	433 (380–577)	100 (96–229)^a^	.06
Time on CMV prior to HFPV, hours	42 (6–109)	43 (6–108)	38 (5–160)	.94
Time on HFPV, hours	124 (77–191)	164 (92–196)	82 (33–117)	.61
Air leak post HFPV	2 (7)	0	2 (29)	.05
VIS pre HFPV	4 (0–11)	3 (0–10)	5 (0–26)	.52
VIS 24 h post HFPV	6 (0–10)	5 (0–10)	6 (1–36)	.44
VIS 48 h post HFPV	4 (0–9)	2 (0–7)	9 (1–17)	.19
Nitric oxide	12 (40)	8 (33)	4 (57)	.39
Continuous neuromuscular blockade during HFPV	29 (94)	23 (96)	6 (86)	.4
Transfer to another institution	10 (32)			
Transfer because for subspecialty need	8 (26)			
Transfer for higher level of hemodynamic support	2 (6)			
Died during hospitalization	5 (16)			

Data are presented as median (interquartile range) or number (%). CMV, conventional mechanical ventilation; HFPV, high-frequency percussive ventilation; LOS, length of stay; PICU, pediatric intensive care unit; VIS, vasoactive infusion score; PIM3, pediatric index of mortality 3.

a Total ventilation time was not calculable for 2 patients in the HFPV-failure group who were transferred for ECMO consideration prior to extubation.

Etiology of respiratory failure: Primary respiratory etiology was present in 55% of patients, including viral pneumonia (10), bacterial pneumonia (4), aspiration pneumonia (2), and asthma (1). Children with non-respiratory primary etiology for respiratory failure included sepsis without respiratory source (6), trauma (3), post-cardiac arrest (2), drowning (1), poisoning (1), and malignancy (1).

Two hours prior to HFPV initiation, median PEEP was 10 cm H_2_O (IQR 8–12), PIP was 32 cm H_2_O (IQR 28–35), and mPaw was 18 cm H_2_O (IQR 16–20), with no significant differences between groups. At 2 h after HFPV initiation, the percussive rate was higher in the success group (600 [550–687]) compared with the failure group (500 [350–600]; *P* = 0.03). No other HFPV settings differed significantly (Table 2). Two patients (7%) developed an air-leak complications during HFPV. Twelve patients (40%) received inhaled nitric oxide, and 94% received continuous neuromuscular blockade.

**Table 2:** Invasive mechanical ventilation settings before and after transition to high-frequency percussive ventilation.

Variable	All	Success	Failure	*P* value
Conventional mechanical ventilation settings 2 h pre HFPV				
PEEP, cmH_2_O	10 (8–12)	10 (9–12)	8 (7.5–11)	.09
PIP, cmH_2_O	32 (28–35)	31 (28–35)	34 (27–40)	.55
mPAW, cmH_2_O	18 (16–20)	18 (17–19)	17 (15–22)	.77
Rate, breaths/min	21 (18–24)	19 (15–24)	23 (22–24)	.24
HFPV settings 2 h after initiation				
PIP, cmH_2_O	36 (29–41)	37 (28–41)	31 (29–47)	.72
PEEP, cmH_2_O	13 (10–16)	13 (10–16)	14 (10–22)	.41
mPAW, cmH_2_O	22 (20–27)	22 (20–26)	23 (20–29)	.76
Convective rate, breaths/min	18 (16–23)	17 (15–23)	19 (18–23)	.34
Percussive rate, cycles/min	600 (500–635)	600 (550–687)	500 (350–600)	.03

Data are presented as median (interquartile range). PEEP, positive end-expiratory pressure; PIP, peak inspiratory pressure; mPAW, mean airway pressure; HFPV, high-frequency percussive ventilation.

**Figure 1:**
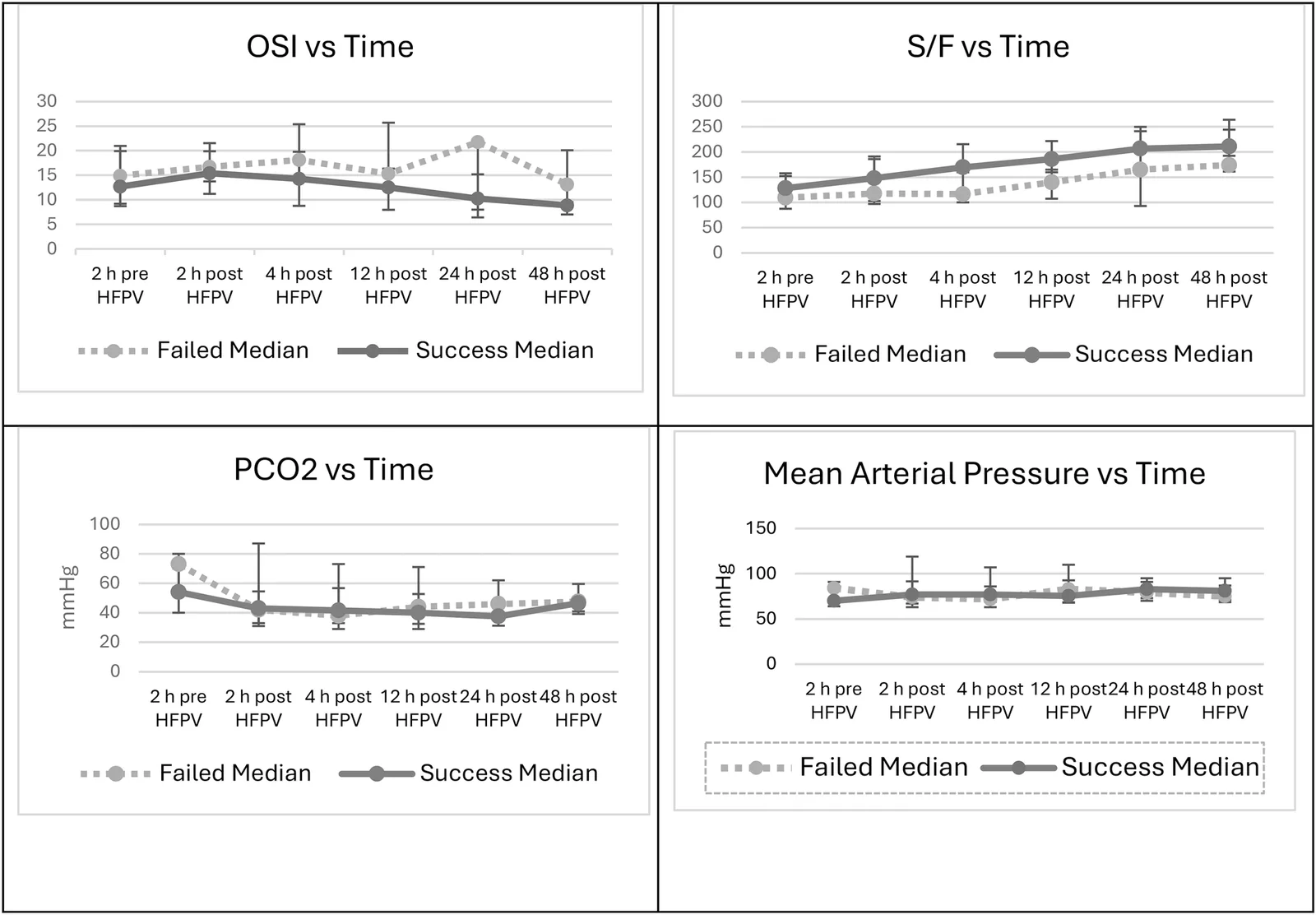
Temporal trends in oxygenation and physiologic parameters following initiation of high-frequency percussive ventilation (HFPV). Median oxygen saturation index (OSI), SpO_2_/FIO_2_ ratio (S/F), partial pressure of carbon dioxide (PCO_2_), and mean arterial pressure (MAP) are shown at predefined time points 2 h before and 2, 4, 12, 24, and 48 h after HFPV initiation, stratified by HFPV success and failure. Error bars represent interquartile ranges. OSI, oxygen saturation index; S/F, oxygen saturation to fraction of inspired oxygen ratio; PCO_2,_ partial pressure of carbon dioxide; MAP, mean arterial pressure.

Among the 26 patients with available pre-HFPV OSI data, 9 (35%) met criteria for mild-to-moderate and 17 (65%) met criteria for severe PARDS based on OSI-based severity groups (13). Oxygenation trajectories differed between the success and failure groups, with higher OSI values in the failure group compared with the success group at 24 h (21.7 [IQR 6.4–22.0] vs. 10.3 [IQR 8.0–15.2]) and lower S/F ratios in the failure group at 24 h (165.0 [IQR 92.8–250.0] vs. 206.7 [IQR 163.5–241.3]) (Figure 1). VIS remained stable across time points: 4 (IQR 0–11) pre-HFPV, 6 (IQR 0–10) at 24 h, and 4 (IQR 0–9) at 48 h, with no significant differences between groups.

ROC analysis was used to evaluate the predictive ability of delta-OSI at multiple time points following HFPV initiation (Table 3). Of the 31 patients in the cohort, 5 lacked a baseline OSI due to a missing mean airway pressure on conventional mechanical ventilation. An additional 2 patients lacked a recorded mean airway pressure at the 2-h post-HFPV time point, and 1 patient died at approximately 25 h after HFPV initiation and therefore lacked a 48-h value. Because the combined ROC model required complete delta-OSI data at all five time points, these 8 patients were excluded, yielding a final analytic sample of 23 patients (19 with HFPV success and 4 with HFPV failure), for a failure rate of 17% among patients with complete data across all time points. The AUC increased progressively from 4 to 24 h after HFPV initiation, with the highest discriminative performance at 24 h.

**Table 3:** Receiver operating characteristic (ROC) curve analysis of oxygen saturation index (OSI) at predefined time points following initiation of high-frequency percussive ventilation.

Variables	2 h (*n* = 23)	4 h (*n* = 23)	12 h (*n* = 23)	24 h (*n* = 23)	48 h (*n* = 23)
AUC (CI)	0.539 (0.223–0.856)	0.697 (0.479–0.916)	0.737 (0.535–0.939)	0.829 (0.649–1.009)^a^	0.737 (0.509–0.964)
*P* value	.807	.077	.022	.001	.041
Max Youden's index	0.276	0.526	0.684	0.684	0.487
Delta-OSI at Youden index	−0.88	0.2	−1.8	1.64	5.58
Sensitivity	0.53	0.53	0.68	0.68	0.47
Specificity	0.75	1.0	1.0	1.0	1.0

AUC, area under the curve; CI, confidence interval. Delta-OSI, Pre-HFPV OSI—Post-HFPV OSI at various time points. HFPV success was treated as the positive outcome in all ROC analyses, with higher delta-OSI values associated with success and lower or negative values associated with failure.

a The upper bound of the 24-h AUC confidence interval nominally exceeds 1.0 due to the use of the asymptotic method for confidence interval estimation in a small sample with an AUC near the ceiling; the true value is bounded at 1.0.

## Discussion

In this retrospective cohort study of pediatric patients with acute respiratory failure managed with HFPV, we found that oxygenation parameters, particularly OSI, showed exploratory discrimination between HFPV success and failure, particularly at 24 h after HFPV initiation. To our knowledge, this is the first report evaluating HFPV outcomes for pediatric acute respiratory failure in a PICU without extracorporeal membrane oxygenation capability, a setting in which early identification of patients at risk of failure is critical, given the time-sensitive nature of coordinating transfer to an ECMO-capable center.

Our study suggests that changes in oxygenation, measured by OSI changes from baseline, where associated with our definitions of HFPV success and failure as early as 12 h after initiation, with the association predictive performance observed at 24 h. Prior pediatric studies have consistently demonstrated improvements in oxygenation following transition to HFPV, often within the first 6–12 h (6, 8, 14). Butler et al. evaluated gas exchange at 2 h after HFPV initiation and found that pH, PaCO_2_, and oxygenation index at that early time point were not associated with mortality; however, after adjustment for confounders, these same variables measured at 2 h were independently associated with HFPV success, defined as improvement or successful transition off HFPV to another ventilatory mode or extubation (7). In contrast, our study incorporated a longer observation period, allowing us to capture clinically meaningful oxygenation trajectories associated with HFPV success or failure.

In our cohort, HFPV failure occurred in 23% of patients, with an overall mortality of 16%, rates comparable to previously published pediatric HFPV series involving heterogeneous populations with acute respiratory failure (6). Butler et al. reported a mortality rate of 22% in the largest pediatric HFPV cohort to date, which included a heterogeneous and severely ill population with high PRISM III scores (7). In contrast, when HFPV is applied in more homogeneous populations with primary respiratory disease, such as bronchiolitis, reported mortality has been substantially lower (8). Our cohort had a heterogeneous population with 55% of patients having primary respiratory pathology. In our cohort, two patients required transfer to outlying facilities for ECMO evaluation, consistent with prior pediatric studies reporting escalation to ECMO in approximately 4.5%–6% of patients receiving HFPV (6, 8, 14).

Vasoactive infusion scores remained stable throughout HFPV support in our cohort. Similar VIS trends have been described in other pediatric HFPV series (6, 7, 14). It is worth noting, however, that a stable median VIS does not exclude the possibility of hemodynamic effects at the individual patient level, and differences in underlying diagnoses, illness severity, and concurrent therapies across these cohorts limit direct comparison.

A key strength of this study is that it reflects the use of advanced modes of ventilation, such as HFPV, in small community PICUs without ECMO capability, which represent a substantial proportion of PICUs in the United States (15) whereas most published pediatric HFPV data originate from large academic centers (6–8). Although PICU bed capacity and ECMO utilization are increasing nationally, this growth has occurred predominantly within established medium- and large-sized PICUs (15–17). Consequently, children in under-resourced or rural regions are less likely to receive care in ECMO-capable centers, contributing to disparities in access to life-saving cardiopulmonary support (18). Together, these factors highlight the importance of generating evidence from smaller PICUs and developing guidance to support the management of patients requiring advanced modes of ventilation, the early identification of treatment failure, and timely transfer to ECMO-capable centers.

Additional strengths of this study include a comprehensive chart review that enabled detailed characterization of patient trajectories, and the use of prespecified, time-based data collection, which enhanced consistency and reduced potential bias in physiologic trend analysis. These features strengthen the relevance of our findings to similar resource-limited pediatric intensive care settings.

This study has several limitations. First, its retrospective, single-center design without a comparison group limits the generalizability of our findings to institutions with different patient populations or practice patterns. Second, the absence of a standardized ventilator management protocol and reliance on individual clinician discretion introduced variability in how HFPV was applied and adjusted. Third, most blood gas measurements were venous, limiting the availability of PaO_2_; however, oxygenation was effectively assessed using validated SpO_2_-based indices such as OSI and the S/F ratio. Fourth, excluding patients on HFPV for less than 12 h introduces the possibility of selection bias, and bundling death with transfer to another institution into a single outcome may mask clinically important differences between these groups. Fifth, because data availability at later time points depended on how long each patient remained on HFPV, missingness was not random and may have differentially affected comparisons across time points. Sixth, SpO_2_-based indices such as OSI and the S/F ratio require adequate peripheral perfusion and pulse-oximeter signal quality, which were not systematically documented in the electronic medical record. Finally, the small sample size, particularly in the failure group, reduces statistical power and increases the risk of type II error.

Our findings suggest that a lack of improvement in OSI within the first 12–24 h of HFPV initiation may aid early clinical decision-making, such as prompting closer monitoring or earlier consultation with ECMO-capable institutions. SpO_2_-based indices such as OSI are widely available at the bedside and supported by current guidelines, making them practical tools for routine monitoring. These data may inform the development of clinical frameworks to help differentiate between likely success and failure of HFPV, guiding ECMO-capable centers on escalation timing and assisting non-ECMO centers in determining when to initiate discussions with referral facilities.

Future prospective multicenter studies are needed to validate OSI thresholds and optimal time points for risk stratification during alternative modes of ventilation such as HFPV. Further investigation into protocol-driven strategies for HFPV initiation and titration, as well as standardized criteria for weaning or defining treatment failure, may improve consistency in care delivery. Ultimately, the early OSI trajectory may hold promise as a tool for identifying patients at risk of HFPV failure, which, if validated, could facilitate timely intervention and potentially improve outcomes in pediatric acute respiratory failure.

## Data Availability

The raw data supporting the conclusions of this article will be made available by the authors, without undue reservation.
