# Supplementary material for: High-frequency percussive ventilation in pediatric acute respiratory failure in a community-based pediatric intensive care unit
Source: Front Pediatr. 2026 Sep 11;14:1864661. doi: 10.3389/fped.2026.1864661 (PMC13612426; doi:10.3389/fped.2026.1864661)
Supplement: Supplementary file 1 [file Table1.docx]

## **Supplementary**

**Table S1.** Details of HFPV failure events

| **Case** | **Reason for Transfer / Cause of Death** | **Last Recorded OSI** | **Hospital LOS, hours** |
| --- | --- | --- | --- |
| *Transfers to Another Institution (n=2)* | | | |
| 1 | Escalation of care, ECMO consult | 13.6 | 118.8 |
| 2 | Escalation of care, ECMO consult | 20.0 | 233.6 |
| *Deaths During Hospitalization (n=5)* | | | |
| 3 | Traumatic brain injury with subarachnoid hemorrhage, cardiogenic shock. | 3.3 | 95.7 |
| 4 | Cardiac arrest secondary to tumor lysis syndrome after chemotherapy | 20.4 | 295.5 |
| 5 | B. pertussis pneumonia complicated by pulmonary hypertension and hyperleukocytosis | 12.7 | 237.0 |
| 6 | Septic shock secondary to candida; PARDS | 11.6 | 1,239.8 |
| 7 | Cardiac arrest complicated by cerebral edema and acute kidney injury | 21.7 | 30.3 |

OSI = oxygen saturation index; LOS = length of stay; ECMO = extracorporeal membrane oxygenation; PARDS = pediatric acute respiratory distress syndrome. OSI values reflect the last predefined data-collection time point before death or transfer (2 hours before and 2, 4, 12, 24, and 48 hours after HFPV initiation). For patients whose ventilation time or hospital stay extended beyond 48 hours, the 48-hour post-HFPV value is reported, as no later time points were collected."
